# Abnormal Bone Collagen Cross‐Linking in Osteogenesis Imperfecta/Bruck Syndrome Caused by Compound Heterozygous *PLOD2* Mutations

**DOI:** 10.1002/jbm4.10454

**Published:** 2021-01-03

**Authors:** Charlotte Gistelinck, MaryAnn Weis, Jyoti Rai, Ulrike Schwarze, Dmitriy Niyazov, Kit M Song, Peter H Byers, David R Eyre

**Affiliations:** ^1^ Department of Orthopaedics and Sports Medicine University of Washington Seattle WA; ^2^ Department of Laboratory Medicine and Pathology University of Washington Seattle WA; ^3^ Department of Pediatrics Ochsner Hospital for Children New Orleans LA; ^4^ Department of Orthopaedic Surgery, David Geffen School of Medicine UCLA Health Los Angeles CA; ^5^ Departments of Pathology and Medicine (Medical Genetics) University of Washington Seattle WA

**Keywords:** BRUCK SYNDROME, COLLAGEN, LYSYL HYDROXYLASE 2, OSTEOGENESIS IMPERFECTA, *PLOD2*

## Abstract

Bruck syndrome (BS) is a congenital disorder characterized by joint flexion contractures, skeletal dysplasia, and increased bone fragility, which overlaps clinically with osteogenesis imperfecta (OI). On a genetic level, BS is caused by biallelic mutations in either *FKBP10* or *PLOD2*. *PLOD2* encodes the lysyl hydroxylase 2 (LH2) enzyme, which is responsible for the hydroxylation of cross‐linking lysine residues in fibrillar collagen telopeptide domains. This modification enables collagen to form chemically stable (permanent) intermolecular cross‐links in the extracellular matrix. Normal bone collagen develops a unique mix of such stable and labile lysyl‐oxidase–mediated cross‐links, which contribute to bone strength, resistance to microdamage, and crack propagation, as well as the ordered deposition of mineral nanocrystals within the fibrillar collagen matrix. Bone from patients with BS caused by biallelic *FKBP10* mutations has been shown to have abnormal collagen cross‐linking; however, to date, no direct studies of human bone from BS caused by *PLOD2* mutations have been reported. Here the results from a study of a 4‐year‐old boy with BS caused by compound heterozygous mutations in *PLOD2* are discussed. Diminished hydroxylation of type I collagen telopeptide lysines but normal hydroxylation at triple‐helical sites was found. Consequently, stable trivalent cross‐links were essentially absent. Instead, allysine aldol dimeric cross‐links dominated as in normal skin collagen. Furthermore, in contrast to the patient's bone collagen, telopeptide lysines in cartilage type II collagen cross‐linked peptides from the patient's urine were normally hydroxylated. These findings shed light on the complex mechanisms that control the unique posttranslational chemistry and cross‐linking of bone collagen, and how, when defective, they can cause brittle bones and related connective tissue problems. © 2020 The Authors. *JBMR Plus* published by Wiley Periodicals LLC. on behalf of American Society for Bone and Mineral Research.

## Introduction

Bone is largely a highly organized extracellular composite of collagen fibrils (primarily type I collagen) intimately mineralized with hydroxyapatite nanocrystals.^(^ ^1^ ^)^ Although the mineral plays a major role in determining bone stiffness and yield strength, overall bone material properties including ultimate tensile strength and toughness heavily depend on the properties of the fibrillar collagen framework.^(^ ^2^, ^3^ ^)^ Osteoblasts produce the collagen fibrils, which in the mature tissue, are heteropolymers of 97% type I interspersed on a template of 3% type V collagen molecules.^(^ ^4^ ^)^ At the genetic level, *COL1A1* and *COL1A2* genes encode the proα1(I) and proα2(I) chains. In the endoplasmic reticulum (ER), two proα1(I) chains and one proα2(I) chain associate to form the triple helical molecule. The nascent chains are cotranslationally and posttranslationally modified in the ER by enzymes contained in chaperone complexes. These modifications are essential for normal folding, assembly, transport, and specialized cross‐linking of the molecular monomers in the extracellular matrix (ECM).

Lysyl hydroxylation is required for the formation of stable (irreversible) intermolecular cross‐links. All fibrillar collagen types have evolved site‐specific lysyl residues in both helical‐peptide and telopeptide domains that through tissue‐dependent controlled hydroxylation modulate the chemistry of eventual extracellular cross‐link formation. Lysine hydroxylation is catalyzed in vertebrates by three ER resident lysyl hydroxylase genetic variants (LH1, LH2, and LH3). These enzymes, encoded by *PLOD1, PLOD2*, and *PLOD3*, respectively, belong to the larger family of 2‐oxo‐glutarate–dependent enzymes, which differ in their preferred substrate‐sequence specificities. Expression levels of the LH genes vary among tissue types and during tissue development.^(^ ^5^, ^6^, ^7^, ^8^ ^)^ Lysines in the triple helical domain of type I procollagen, and especially cross‐linking lysines, are hydroxylated primarily by LH1. Telopeptide lysines appear to be hydroxylated solely by LH2b, a long splice variant of LH2. LH3 appears not to play a major role in lysyl hydroxylation of type I procollagen, at least in bone, being most active on basement membrane type IV collagens.^(^ ^9^, ^10^ ^)^

The two splice forms from *PLOD2*, LH2a (short) and LH2b (long), differ by the 21 amino acid residues encoded by exon 13A.^(^ ^11^ ^)^ The LH2b variant is expressed in tissues rich in fibrillar collagens. This is consistent with evidence that telopeptide lysyl hydroxylation requires LH2b activity. To what extent LH2b can also hydroxylate lysines in the triple helical domain and the function of the LH2a splice form are unclear.

Cellular mechanisms controlling *PLOD2*/LH2 expression and LH2 activity are responsible for the well‐recognized tissue‐dependent differences in collagen cross‐linking chemistry.^(^ ^8^, ^12^, ^13^ ^)^ In essence, telopeptide lysines when hydroxylated go on to form chemically irreversible cross‐links after lysyl oxidase oxidation, whereas unmodified lysines produce mostly labile intermolecular cross‐links. Abnormal collagen cross‐linking is seen in osteogenesis imperfecta (OI) and Bruck syndrome (BS) through underhydroxylation and overhydroxylation effects on bone collagen telopeptide lysines.^(^ ^14^, ^15^ ^)^ Biallelic and compound heterozygous mutations in *PLOD2* cause BS type 2 (BS2; MIM 609220), characterized by skeletal anomalies and the bone fragility of OI together with congenital contractures of the large joints.^(^ ^14^ ^)^ Biallelic mutations in *FKBP10*, which encodes the ER procollagen chaperone FKBP65, also prevent type I collagen telopeptide lysine hydroxylation, and result in Bruck syndrome type 1 (BS1; MIM259450), which is clinically indistinguishable from BS2. It was recently shown that FKBP65 and LH2 form a complex in the ER that is required for LH2 dimerization and telopeptide hydroxylase activity,^(^ ^16^, ^17^, ^18^ ^)^ so explaining the *PLOD2* and *FKBP10* phenocopies. Early studies on BS patients with BS with *FKBP10* mutations had established that collagen cross‐linking defects were associated with the skeletal phenotype.^(^ ^19^, ^20^ ^)^

In normal human bone collagen, telopeptide lysines are partially hydroxylated (~50%), compared with 0% in skin type I collagen and 100% in cartilage types I and II collagens.^(^ ^1^ ^)^ The mechanism regulating this partial lysyl hydroxylation in bone, which results in a mixture of pyridinoline (Pyr) and pyrrole mature trivalent cross‐links,^(^ ^21^, ^22^ ^)^ is poorly understood. Pyrrole cross‐links are tissue restricted to bone and certain tendons and their presence seems to be correlated with the highly ordered deposition of hydroxyapatite nanocrystals in bone collagen fibrils.^(^ ^1^, ^22^, ^23^ ^)^ In bone from individuals with BS1 the absence of type I collagen telopeptide hydroxylysine results in a lack of pyrrole and Pyr cross‐links and a cross‐linking profile, collagen extractability and electrophoretic pattern that resembles that of skin, not bone.^(^ ^20^ ^)^ In the same study, based on urinary peptide analysis, it was also shown that cartilage type II collagen telopeptide lysines had been normally hydroxylated and incorporated into hydroxy lysyl Pyr cross‐links.

These earlier patient studies and mouse and zebrafish genetic models of BS all point to an association between lack of hydroxylysine aldehyde cross‐linking in bone collagen and bone fragility.^(^ ^17^, ^18^, ^24^, ^25^, ^26^ ^)^ In a Plod2 knockout zebrafish model, telopeptide lysine hydroxylation was blocked and collagen type I was more extractable from bone consistent with a decrease in stable cross‐links.^(^ ^24^ ^)^ This implies that altered collagen cross‐linking might more generally contribute to bone fragility in OI and other conditions. However, the effect of *PLOD2*/LH2 deficiency on human bone has not yet been investigated directly. Here we report a selective effect on collagen cross‐linking in bone but not cartilage of a patient with BS2 caused by compound heterozygous mutations in *PLOD2*, which phenocopies the effect of *FKBP10* mutations causing BS1.

## Patients and Methods

### Clinical information and tissue collection

**Fig 1:**
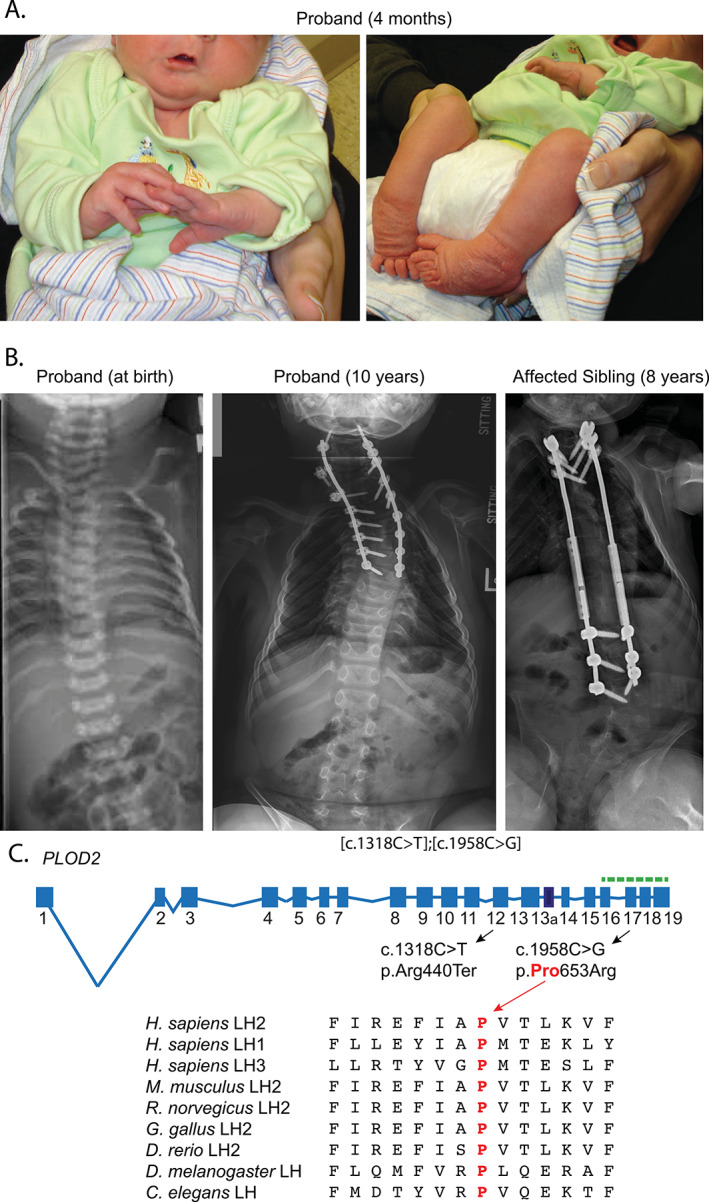
Clinical and molecular studies. (*A*) Photographs taken at age 4 months of the proband showing contractures of the joints. (*B*) Radiographs of proband at birth and 10 years, and of the affected sibling at the age of 8 years. Radiographs of proband show multiple rib fractures and platyspondyly at birth and the fixation of cervical and upper thoracic spine at the age of 10 years. The affected sibling shows scoliosis, partially corrected by surgical rodding at age 8 years. (*C*) Schematic representation of the genomic structure of the *H. sapiens PLOD2* gene. Boxes and lines represent exons and introns, respectively. Exon 13a (thick box border) is subject to differential splicing. The catalytic domain is encoded by the last four exons of *PLOD2* (indicated by the green dashed line). One mutation in the proband results in a stop‐codon in exon 12 of *PLOD2* (p.Arg440Ter), whereas the other allele carries a mutation that substitutes a highly conserved Pro residue in exon 17 of PLOD2 (p.Pro653Arg). The location of this mutation, which is a part of the catalytic domain, represents a mutation hotspot^(^ ^37^, ^38^, ^41^ ^)^; the substitution was predicted to be deleterious in silico (polyPhen). HP = hydroxy lysyl pyridinoline; LP = lysyl pyridinoline.

The proband was born by caesarean section because of a breech presentation at 36‐6/7‐week gestation after an uneventful pregnancy. He was the first child born to unrelated healthy White parents: a 24‐year‐old mother and a 31‐year‐old father. At birth, multiple joint contractures involving the knees, elbows, and ankles, a left‐sided torticollis, and bilateral clubfeet were noted (Fig. 1*A*). These were not seen in ultrasound studies in the few weeks prior to delivery. X‐rays of skull, C‐spine, chest, and lower extremities showed Wormian bones and multiple bilateral healing rib fractures (Fig. 1*B*). He passed the newborn hearing test. His sclerae were white. At age 5 days, a pediatrician considered the diagnosis of BS. An exam at age 8 days also noted a very large anterior fontanelle that extended to the occipital bone.

A follow‐up consultation at age 22 months noted marked improvement of the clubfeet after casting and mobilization of the elbow contractures with physical therapy. He still had pronounced limitations of his knee and hip movement. He had deep dimples on his lateral ankles, knees, and elbows, and there was beginning web formation over his elbows and knees. He had decreased muscle mass that was most obvious in the extremities. He had sustained four fractures in the 4 months before the clinic visit. At age 4 years, he underwent surgery to release the knee contractures; bone fragments from the right femur, a small piece of skin, and a urine sample were saved for additional studies (described below). He had fixation of the cervical and upper thoracic spine at age 6 years (Fig. 1*B*). By age 10, he had sustained 80 to 90 fractures, most in his limbs and ribs. At age 10 years, his weight was 69 lbs (31.6 kg; 50th percentile), height (difficult to measure because of the contractures) 3 ft 7 in. (109 cm). Head circumference was 21 in. 53 cm (50th percentile) with some positional plagiocephaly. He is mostly confined to a wheelchair; he can pull up to stand in his braces but is limited because of his knee contractures, which were released several times but recur. He has mild contractures in both elbows, midfacial hypoplasia, and a prominent forehead.

His younger sister has had 15 to 20 fractures, mainly of the ribs and clavicles. She had more severe scoliosis and required rods for a long part of her spine that took two different procedures (ages 7 and 8 years) because the hardware failed after the first surgery. She had no contractures, and her joints were actually lax (3‐4 points on the Beighton scale). At age 8 years, she weighed 21.3 kg 47 lbs (10th percentile) and height was 114 cm 3′9″ (~5th percentile). Her head circumference was 53 cm 21 in. (50th percentile) with some positional plagiocephaly. She has midfacial hypoplasia and a bulbous nasal tip, as well as prominent forehead with scars on her forehead from previous halo placement for neck surgery. She had a mild micrognathia. She can stand without support and walk holding onto the table. She has better motor function than her brother.

Both have normal language and cognitive development (even precocious). Both children were treated with pamidronate by infusion, according to the Montreal protocol, from shortly after birth through their current ages. Neither parent has bone fragility or other signs compatible with BS. The clinical details are provided with parental consent; all studies were done with appropriate institutional approval and parental consent.

### Mutation identification in *PLOD2*

Genomic DNA was extracted from peripheral blood using the Puregene DNA purification kit (Gentra). The 20 exons of *PLOD2* (*NM_182943.2*) and flanking intronic sequences were amplified in 16 reactions (primer sequences are available on request). Sequencing reactions were done using Big Dye Terminators, version 3.1 (Applied Biosystems), and run on an AB 3130XL Genetic Analyzer. Sequences were analyzed using Mutation Surveyor (Softgenetics).

### Tissue preparation and SDS‐PAGE analysis of bone collagen

A right femur biopsy was obtained from the patient with appropriate consent. Control adult bone was obtained from Northwest Tissues Services in Renton, Washington. Bone was defatted in chloroform/methanol (3:1 v/v) and demineralized in 0.1M HCl for 16 hours at 4°C. Collagen was then solubilized from an aliquot of each tissue preparation by heat denaturation in SDS‐PAGE sample buffer, and the chains were separated in 6% SDS‐PAGE gels.^(^ ^22^ ^)^ Because of the young age of the patient's bone (4.5 years), both normal fetal and adult bone were used as controls with fetal being considered the closest in development for comparison.

### Pyr cross‐link analysis

Bone and urine samples were hydrolyzed in 6M HCl, dried, redissolved in 1% (v/v) n‐heptafluorobutyric acid for analysis by C18 reversed‐phase high‐performance liquid chromatography (RP‐HPLC) using an established procedure to resolve and quantify hydroxy lysyl pyridinoline (HPyr) and lysyl pyridinoline (LPyr) by their inherent fluorescence.^(^ ^27^ ^)^

Liquid chromatography–tandem mass spectrometry of total cross‐linking amino acids

Decalcified bone was reduced with sodium borohydride and hydrolyzed in 6M HCl at 108°C for 24 hours. Cross‐linking amino acids were enriched from hydrolysates by partition on a hydrated cellulose column and analyzed by liquid chromatography‐tandem mass spectrometry (LC‐MS/MS) essentially as originally reported^(^ ^28^ ^)^ and as modified.^(^ ^29^ ^)^ The multicharged cross‐linked amino acids were quantified by electrospray LC‐MS/MS on an LTQ XL using a Cogent 4 diamond hydride column (15 cm × 1 mm; 70000‐15P‐1; Microsolv Technology) eluted at 50 μl/min. The LC mobile phase consisted of buffer A (0.1% formic acid in MilliQ water) and buffer B (0.1% formic acid in 80% acetonitrile).

### MS of collagen peptides

In‐gel trypsin digests of collagen chains cut from SDS‐PAGE gels were carried out as described.^(^ ^30^, ^31^ ^)^ Demineralized bone was digested with bacterial collagenase.^(^ ^22^ ^)^ The resulting collagen‐derived peptides were spread by RP‐HPLC (4.6 mm × 25 cm; C8; Brownlee Aquapore RP‐300) with a linear gradient of acetonitrile:n‐propanol (3:1 v/v) in aqueous 0.1% (v/v) trifluoroacetic acid.^(^ ^31^ ^)^ The Pyr cross‐linked type II collagen C‐telopeptide, previously identified in urine,^(^ ^21^, ^32^ ^)^ was enriched by reversed‐phase and ion‐exchange cartridge extraction and resolved by C8 HPLC for mass spectrometric analysis.

Electrospray MS was performed on in‐gel trypsin digests and individual HPLC column fractions using an LTQ XL ion‐trap mass spectrometer, which was equipped with in‐line LC (Thermo Fisher Scientific) using a C4 5‐μm capillary column (300 μm × 150 mm; Higgins Analytical RS‐15M3‐W045) eluted at 4.5 ml/min. The LC mobile phase consisted of buffer A (0.1% formic acid in MilliQ water) and buffer B (0.1% formic acid in 3:1 acetonitrile:n‐propanol v/v). The LC sample stream was introduced into the mass spectrometer by electrospray ionization with a spray voltage of 4 kV. Proteome Discoverer search software (Thermo Fisher Scientific) was used for linear peptide identification using the National Center for Biotechnology Information protein database. Cross‐linked peptides and glycosylated variants were identified manually by calculating theoretical parent ion masses and possible MS/MS ions and matching these to the actual parent ion mass and MS/MS spectrum.

## Results

### Identification of compound heterozygous *PLOD2* mutations in both children

Two pathogenic variants were identified in both siblings: c.1318C>T, p.Arg440Ter, and c.1958C>G, p.Pro653Arg (Fig. 1*C*). Mutation analysis of parental DNA showed a trans configuration (mutations on separate alleles) consistent with recessive inheritance. The first resulted in a premature termination codon in exon 12, which is expected to result in mRNA instability and no active protein from that allele. The second alters a residue in exon 17 in the active site region of the enzyme and has been previously identified in a different individual with BS in the context of a premature termination codon in the second allele (unpublished results, Peter H Byers).

### Effect of *PLOD2* mutations on the content of HPyr and LPyr cross‐links in bone and urine

Collagen molecules in fibrils are linked covalently by intermolecular lysyl (Lys)– and hydroxy lysyl (Hyl)–derived cross‐links. The mature cross‐links include Pyrs, which link three chains that are in three different collagen molecules. Their precursors are two telopeptide Hyl residues and a Lys (LPyr) or Hyl (HPyr) residue from a triple helix. Normal human bone collagen has a distinctive content of Pyr and ratio of HPyr:LPyr, reflecting the partial hydroxylation of both telopeptide and helical site lysines.^(^ ^33^ ^)^ The total content of Pyrs in the patient's bone collagen was 2% of that in control bone with a reversed ratio of HPyr:LPyr (Table 1).

**Table 1:** Pyridinoline Cross‐Links in Bone Collagen

	Ratio HPyr:LPyr	Moles/mole collagen	
		HPyr	LPyr
Patient	0.36	0.0012	0.0034
Control range (age 5–7 y)^(^ ^43^ ^)^	3.5–4.3	0.07–0.16	0.02–0.05

The content of hydroxylysyl pyridinoline (HPyr) and lysyl pyridinoline (LPyr) in collagen was measured in a hydrolysate of the patient's bone.

**Fig 2:**
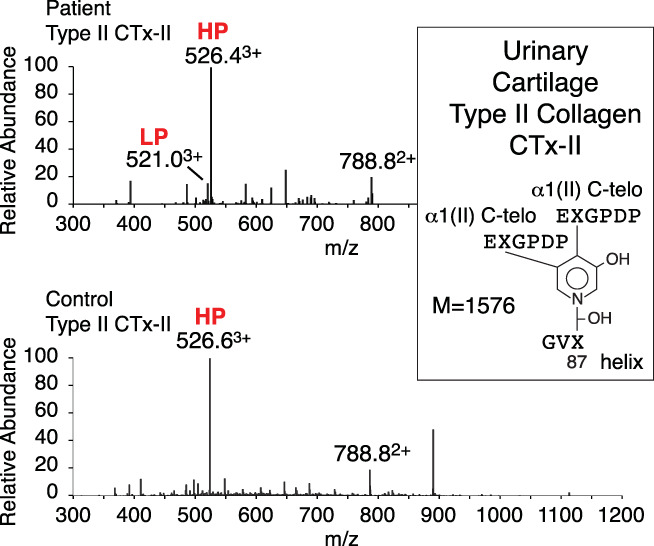
Mass‐spectral analysis of a type II collagen cross‐linked peptide from urine. Patient's urine was fractionated and analyzed by liquid chromatography‐mass spectrometry. Shown is a peptide identified in this sample, which comes from a site of α1(II) C‐telopeptide pyridinoline cross‐linking (M = 1576), reported earlier to be present in control children's urine.^(^ ^20^ ^)^ The parent ion envelope of this HPyr‐containing structure is shown, whereas the posttranslational variant LPyr‐containing cross‐link (486.6^3+^) is only present in very low amounts. This result shows that in contrast to bone type I collagen, the patient's cartilage type II collagen had apparently normal telopeptide lysine hydroxylation, and the low level of the LPyr form of the peptide indicates almost complete hydroxylation of the triple‐helical domain lysines in tissue from which the peptides originated.

Urine also can provide a useful sampling of resorbed bone and growth‐plate collagen peptides for assessing systemic skeletal tissue collagen quality. Cross‐linked peptides excreted in the patient's urine were resolved by HPLC and analyzed by MS. Consistent with the results obtained by analysis of bone, Pyr cross‐linked peptides from type I collagen were barely detectable. The main source of HPyr in the urine was peptides derived from cartilage type II collagen. The main structure (Fig. 2) was the same peptide from the patient's and a growing normal child's urine.^(^ ^21^ ^)^ The peptide is derived from and provides a measure of growth‐plate cartilage resorption. The ratios of HPyr:LPyr in the peptide were similar for both the normal child's and the patient's urine. This indicates that, unlike bone, cartilage type II collagen cross‐linking and telopeptide‐ and helical‐domain lysyl hydroxylation were unaffected by the *PLOD2* mutations.

### SDS‐PAGE analysis of extracted bone collagen

**Fig 3:**
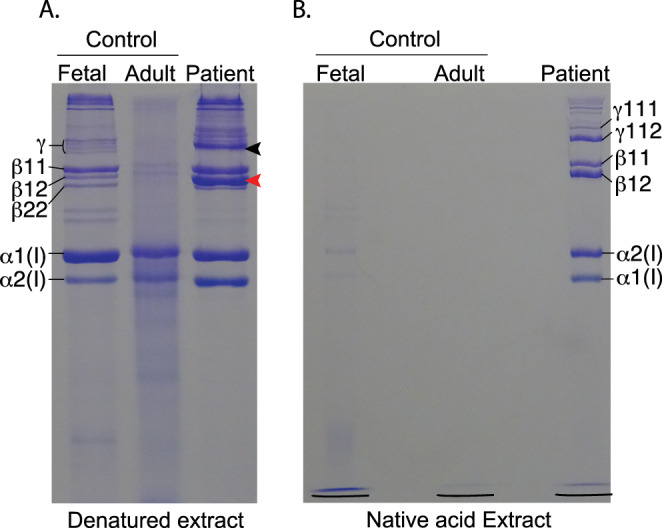
SDS‐PAGE analysis of bone collagen. (*A*) Collagen extracted from fetal control, adult control, and patient's bone was denatured and run on SDS‐PAGE. The pattern of collagen chains extracted from patient's bone is more similar to that of normal skin than bone collagen, with more prominent β‐dimers (red arrowhead) and γ‐trimers (black arrowhead). This is consistent with cross‐linking by lysine aldehydes not hydroxylysine aldehydes. (*B*) Nondenaturing extraction in dilute acetic acid solubilizes little collagen from normal fetal or adult demineralized bone, whereas the patient's bone collagen is highly extractable, consistent with a low level of acid‐stable intermolecular cross‐links.

SDS‐PAGE analysis of type I collagen extracted from the patient's bone shows a chain migration pattern similar to that of skin not bone collagen, with prevalent β‐dimers and γ‐trimers (Fig. 3*A*). The β‐dimers of skin collagen are the result of intramolecular aldol cross‐linking between allysines in α1(I)‐α1(I) and α1(I)‐α2(I) N‐telopeptides. The chain pattern from the patient's bone is consistent, therefore, with a failure of the telopeptide hydroxy lysyl‐dependent aldehyde cross‐linking, which normally operates in bone. Whereas normal bone collagen resists 3% acetic acid extraction because of acid‐stable intermolecular cross‐links, the patient's bone collagen was more extractable (Fig. 3*B*), which is consistent with a decrease in stable intermolecular cross‐links caused by the *PLOD2* mutations.

### Confirmation of divalent aldol cross‐links in *PLOD2* mutant bone collagen

**Fig 4:**
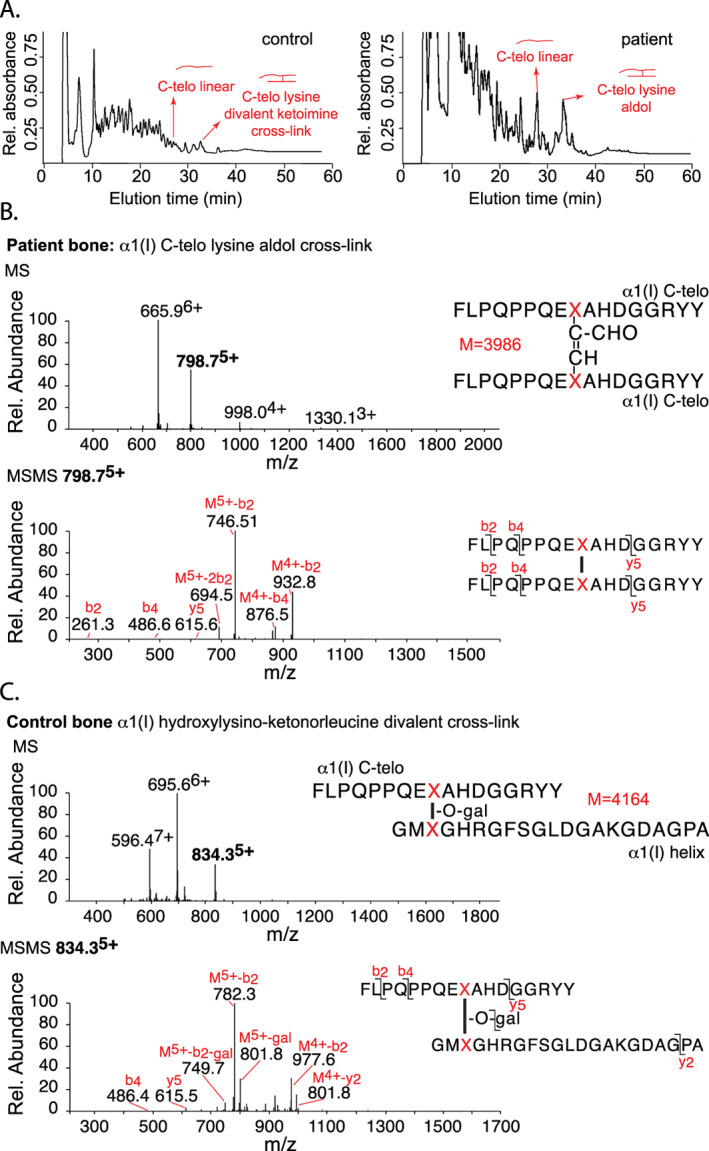
Mass‐spectral analysis of the effect of the *PLOD2* defect on collagen I cross‐linking. Liquid chromatography‐mass spectrometry (LC‐MS) fractionation of collagenase‐digested bone collagen peptides from normal control bone and patient's bone. (*A*) The chromatogram of the patient's bone reveals an increase in the presence of a linear fragment corresponding to the C‐telopeptide of type I collagen (C‐telo linear, confirmed by MS), and an allysine aldol cross‐linked C‐telopeptide dimer (C‐telo lysine divalent cross‐link), which is a minor component of normal bone. (*B*) MS confirmed the peptide dimer structure matched the mass of an allysine aldol cross‐link between two α1(I) C‐telopeptides (structure shown, M = 3986 Da). The parent ion envelope of the latter (665.9^6+^, 798.7^5+^, 998.0^4+^, etc) is evident in the MS spectrum, and the MS/MS fragmentation pattern shown below confirms the two peptide sequences present. (*C*) As a reference, LC‐MS of a digest of normal bone collagen shows in contrast a prominent divalent cross‐linking structure formed between the α1(I) C‐telopeptide of one molecule and the α1(I) helical domain of another molecule, in which the cross‐link has the mass of the galactosyl form of hydroxylysino‐ketonorleucine. The charge envelope of the parent ion (M = 4164), and the MS/MS fragmentation pattern shown below, confirms the structure, which is the precursor for trivalent pyridinoline and pyrrole cross‐links in bone collagen.

The effect on cross‐linked peptides was examined by digesting demineralized bone collagen with bacterial collagenase; the peptides were analyzed by LC‐MS/MS. This gave high yields of the monomer and cross‐linked dimer C‐telopeptide from the patient's bone collagen in contrast to low yields from normal control bone (Fig. 4*A*,*B*). The α1(I)‐C‐telopeptide dimer proved by mass to be cross‐linked by an α,β unsaturated aldol formed between the two lysine aldehyde residues. This same peptide is prominent in digests of normal skin but not bone collagen.^(^ ^33^ ^)^

**Fig 5:**
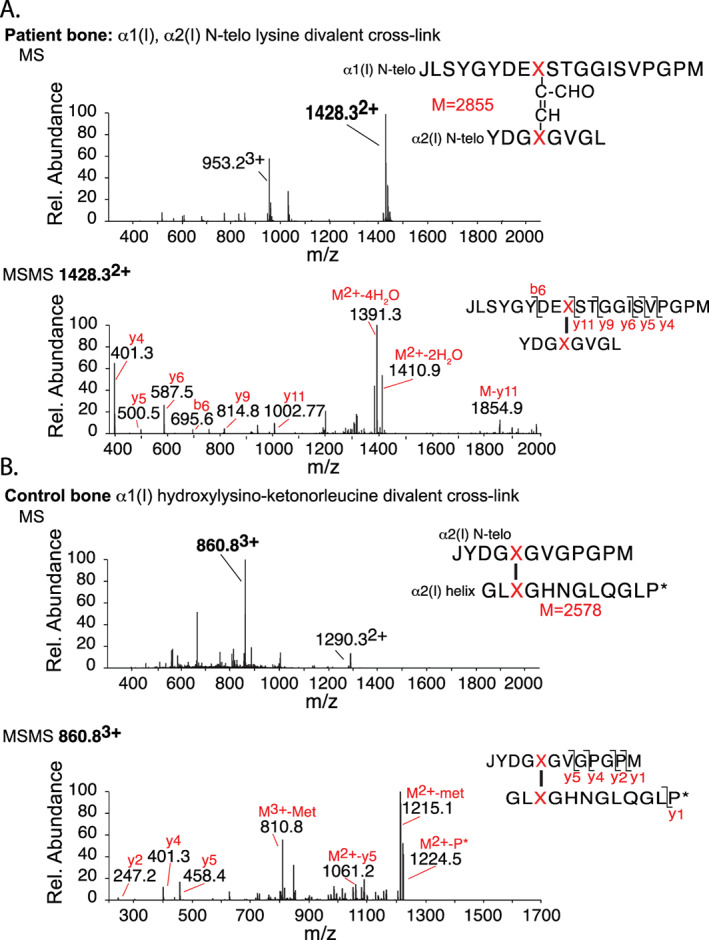
Mass‐spectral identification of N‐telopeptide aldol cross‐linked structures in the patient's bone. (*A*) From fraction 36 of the chromatogram of the collagenase‐digested patient's bone collagen shown in Fig. 4*A* (right panel), we also recovered on liquid chromatography‐mass spectrometry (LC‐MS) analysis a prominent aldol‐cross‐linked α1(I)N to α2(I) N‐telopeptide dimer. Upper panel: The MS spectrum shows the parent ion envelope for M = 2855, which is the mass of the structure shown. Lower panel: The parent ion MSMS fragmentation pattern confirms the structure. (*B*) In contrast with normal bone, the equal mix of hydroxylysine and lysine aldehydes produces divalent keto‐imine intermolecular cross‐links that can mature to trivalent pyridinolines and pyrroles. The peptide shown was prominent on LC‐MS analysis of bacterial collagenase‐digested control bone matrix (Fig. 4*A*, left panel). It originates from a divalent intermolecular hydroxylysino‐ketonorleucine cross‐link between an α2(I) N‐telopeptide and α2(I) K933. Upper panel: The MS spectrum shows the parent ion envelope for M = 2578, the mass of the structure containing this cross‐link. Lower panel: The parent ion MSMS fragmentation pattern confirms the peptide structure.

The analogous aldol dimer formed between α1(I) and α2(I) N‐telopeptides was also recovered from the bacterial collagenase digest of the patient's bone collagen (Fig. 5*A*). Normal bone collagen in contrast reveals prominent divalent keto‐imine cross‐linked peptides on bacterial collagenase digestion as shown for reference (Figs. 4*C* and 5*B*). The latter are the divalent initial cross‐linking products on the hydroxy lysyl aldehyde pathway, some of which go on to interact with each other or the related aldimines from lysyl aldehydes to form stable trivalent Pyr and pyrrole cross‐links, respectively.^(^ ^34^ ^)^ The abnormally high levels of aldol cross‐linked peptides recovered from the patient's bone collagen are consistent with the abundance of β11 dimers and γ112 trimers seen on SDS‐PAGE of the extracted collagen (Fig. 3*A*).

### Analysis of total collagen cross‐links confirms a profile resembling that of tendon or skin not bone

**Fig 6:**
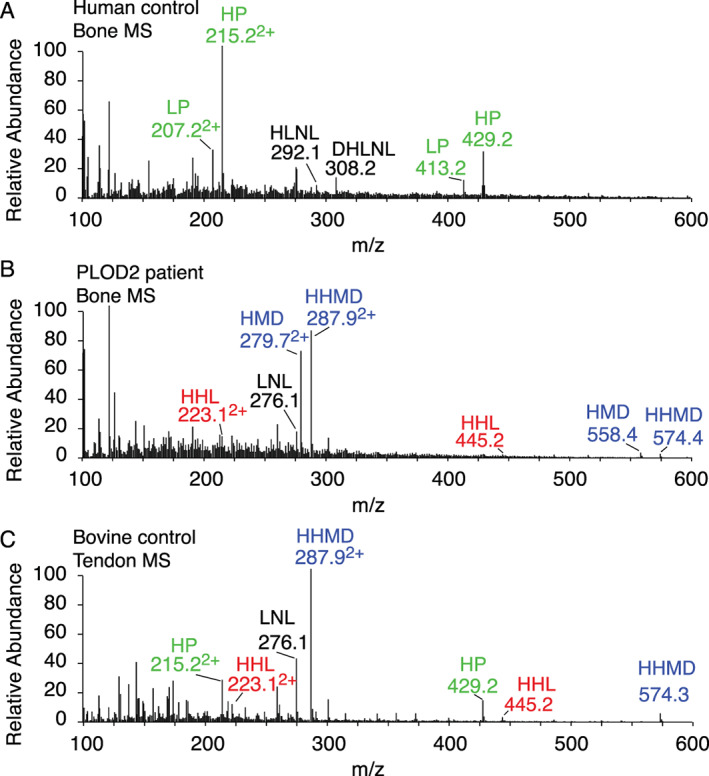
Liquid chromatography‐mass spectrometry (LC‐MS) analysis of collagen cross‐link derivatives in acid hydrolysates of borohydride‐reduced PLOD2‐mutant bone compared with control bone and tendon. (*A*) From control bone, the pyridinoline (Pyr) cross‐links (green) and their divalent precursors, DHLNL and HLNL, predominate. From PLOD2‐mutant bone, Pyrs are absent and histidinohydroxymerodesmosine (HHMD; and histidinomerodesmosine [HMD]) predominate with small amounts of histidinohydroxylysinonorleucine (HHL). (*B*) HHMD and HHL are more typical of skin collagen and are the artifactual products of allysine aldol telopeptide dimers on acid hydrolysis (HHL from C‐telopeptide aldols) and borohydride reduction (HHMD from N‐telopeptide aldols).^(^ ^29^ ^)^ The lysyl homolog of HHMD (HMD) is prominent in *PLOD2*‐mutant bone collagen because the helical‐domain cross‐linking lysines at α1(I)K930 and α2(I)K933 are only partially hydroxylated in normal and *PLOD2* bone. (*C*) For comparison, results are shown from bovine tendon collagen, a tissue that also expresses a mix of telopeptide lysine and hydroxylysine aldehyde cross‐links.

Figure 6 depicts the comparison of the mass spectra of cross‐linking amino acids recovered from normal bone and *PLOD2*‐mutant bone with bovine tendon as a control. HHMD (histidinohydroxymerodesmosine) and HMD (histidinomerodesmosine) are the products of borohydride reduction of N‐telopeptide aldols in aldimine linkage to hydroxylysine (HHMD) and lysine (HMD) at K930 in α1(I).^(^ ^29^ ^)^ HHL (histidinohydroxylysinonorleucine) is an artifactual product on acid hydrolysis from the C‐telopeptide aldol dimer linked to glycosylated K87 in α1(I).^(^ ^29^ ^)^ These results confirm the absence of Pyrs, prominent in normal bone (Fig. 6*A*), and show a similarity to tendon (Fig. 6*C*; published results on skin^(^ ^29^ ^)^). The prominence of HMD in the *PLOD2*‐mutant bone reflects the degree of underhydroxylation of α1(I)K930, which is a characteristic posttranslational feature of normal bone collagen (in which it results in LPyr not HMD).

### Direct evidence of telopeptide lysine underhydroxylation in *PLOD2*‐mutant bone collagen

**Fig 7:**
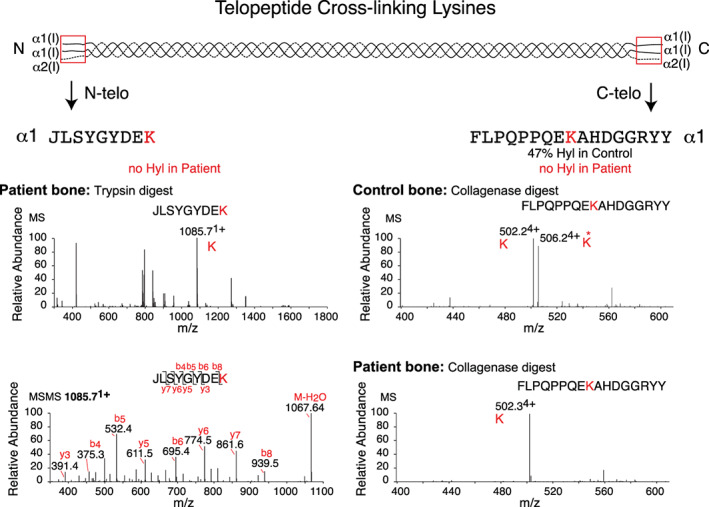
Hydroxylation status of telopeptide cross‐linking lysine residues in type I collagen. Linear (uncross‐linked) peptides from the N‐ and C‐telopeptide domains of collagen α‐chains were prepared by in‐gel trypsin digestion (N‐telopeptide) and bacterial collagenase digestion (C‐telopeptide) of decalcified bone collagen and identified by liquid chromatography‐mass spectrometry (LC‐MS). Right panels: Informative peptides recovered from control bone (upper) and the patient's (lower) bone showed 47% and 0% hydroxylation, respectively. Left panels: The patient's bone yielded only an unhydroxylated α1(I) N‐telopeptide; upper: MS of parent ion; lower: MSMS fragment identification. See Table 2 for yields. No informative α2(I)N‐telopeptide was recovered.

Type I collagen telopeptide domain lysyl residues (K), α1(I)N‐K9, and α2(I)N‐K7 in the N‐telopeptides and α1(I)C‐K16 in the C‐telopeptides, are substrates for LH2 and subsequently lysyl oxidase. We prepared peptides for MS by bacterial collagenase digestion of demineralized bone matrix, or in‐gel trypsin digestion of extracted α chains, to assess the hydroxylation status of these specific lysine residues. This sub‐fraction of linear peptides escaped lysyl oxidase oxidation and hence cross‐linking, and so presumably reflected the hydroxylation status of these sites at synthesis. Results are shown for the α1 telopeptide cross‐linking sites (Fig. 7, Table 2). Despite several attempts, no informative linear peptide could be recovered from the α2 N‐telopeptide. The high yield of the α1, α2 N‐telopeptide aldol dimer (Fig. 5) suggests that this reflects complete oxidation to aldehyde and dimer formation of the (nonhydroxylated) lysyl form of the α2 N‐telopeptide by lysyl oxidase.

**Table 2:** Summary of the Hydroxylation Status of Key Residues in Type I Collagen From the Patient's Compared With Control Bone

Amino acid residue		% of lysine residue hydroxylated		
		Control bone		*PLOD2* patient bone
		Adult	Fetal	
Helical cross‐linking lysines	α1(I) K87	>95	>95	>95
	α1(I) K930	40	60	34
	α2(I) K87	69	83	64
	α2(I) K933	78	77	86
Telopeptide cross‐linking lysines	α1(I) N‐telo K	46	50	<3
	α2(I) N‐telo K	78	62	nd
	α1(I) C‐telo K	66	83	<3
Prolyl 3‐hydroxylation sites	α1(I) P986	96–99	96–99	>95
	α2(I) P707	80	84	73

Linear cross‐linking lysine‐containing peptides from the N‐ and C‐telopeptide domains and the helical domain of collagen α‐chains were prepared by in‐gel trypsin digestion or bacterial collagenase digestion of decalcified bone from the patient and control and identified by liquid chromatography‐mass spectrometry (LC‐MS). MS analysis of the cross‐linking lysine residues in α1(I) is depicted in detail in Figs. 5 and 7. The peptide positions are located with respect to the first glycine of the triple helical domain. In this context, the first glycine in the triple helical domain of proα1(I) chains is p.Gly168 and for proα2(I) chains is p.Gly91.

nd = no linear α2(I)N‐telopeptide detected on LC‐MS.

### Cross‐linking lysines in the triple‐helical domain

**Fig 8:**
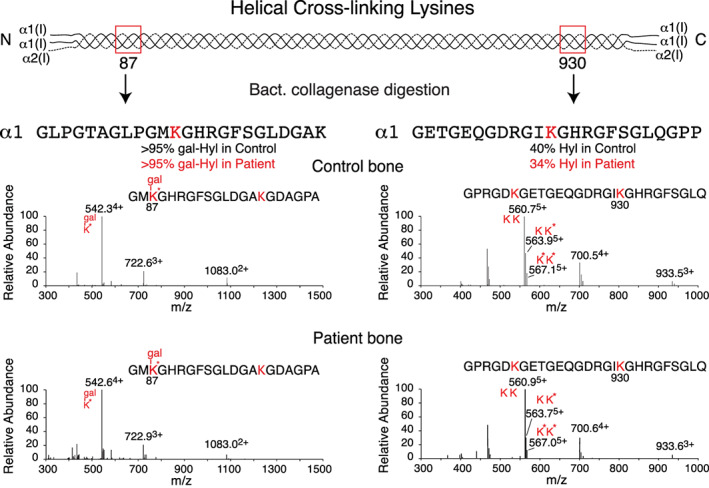
Hydroxylation status of triple‐helical domain cross‐linking lysine residues in type I collagen. Linear (uncross‐linked) peptides from the four molecular sites of cross‐linking in the helical domain of collagen type I were also prepared for liquid chromatography‐mass spectrometry (LC‐MS) analysis by bacterial collagenase digestion of decalcified bone. Results are shown on linear sequences containing residues K87 and K930 from the α1(I)‐chain. The K87 site was 95% hydroxylated from both the normal control's and the patient's bone, of which most was glycosylated primarily as K * gal for both. The K930 site was 40% hydroxylated in control bone and 34% in the patient's bone with no glycosylation (ie, both in the normal bone range). This peptide has two hydroxylatable lysines so the parent ion +16 ladder reflects their status shown as KK, KK*, and K*K*. The hydroxylation percentages in the figure refer specifically to residue K930, based on validation by MSMS fragmentation of the individual parent ions (not shown). See Table 2 for a summary of hydroxylation levels at all helical cross‐linking Lys sites.

Each chain in the triple‐helical domain of type I collagen has two sites to which α1(I) and α2(I) telopeptide Lys/Hyl aldehydes can cross‐link, so there are six potential initial divalent cross‐linked peptides (3 × 2) per molecule. These lysines (α1/α2K87, α1K930, and α2K933, numbered with respect to the first glycine of the triple helical domain) are primarily hydroxylated by LH1, encoded by *PLOD1*. To determine whether LH2 deficiency affected their hydroxylation we examined relevant linear peptides in bacterial collagenase digests of the patient's bone collagen (Fig. 8). Given the variability with age, no significant effect on lysyl hydroxylation at these sites in *PLOD2* bone can be concluded.

For completeness, we also assessed the status of prolyl‐3 hydroxylation at the two principal triple‐helical sites: α1(I)P986 and α2(I)P707. These residues are hydroxylated by the ER‐resident complex, P3H1/CypB/CRTAP. Mutations in any one of the three genes encoding this complex have been shown to alter collagen lysyl hydroxylation and cross‐linking,^(^ ^35^, ^36^ ^)^ so a reverse effect was a possibility. Mass spectral analysis of peptides containing these sites from in‐gel digests of extracted collagen‐α chains showed no significant effect on prolyl‐3 hydroxylation from the patient's bone (Table 2).

**Fig 9:**
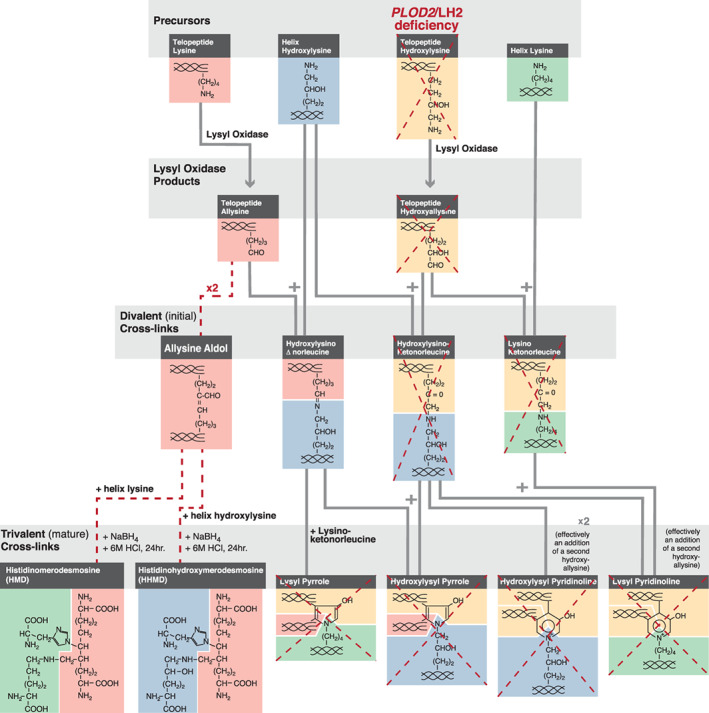
Schematic of the mixed function cross‐linking from lysine and hydroxylysine aldehydes in normal bone collagen to illustrate the effect of lysine aldehydes alone in *PLOD2* bone. In normal human bone type I collagen, approximately 50% of the telopeptide lysines (in red) are converted by *PLOD2*/LH2 to telopeptide hydroxylysines (in yellow). Both are substrates for lysyl oxidase (LOX)–producing reactive aldehydes (allysines) extracellularly, which react with the specific lysine or hydroxylysine side chains (in green and blue respectively) in triple‐helical domains of adjacent molecules forming keto‐imine and aldimine divalent cross‐links. A fraction of the latter goes on to interact, forming permanent trivalent cross‐links (pyrroles and pyridinolines). From 50% telopeptide hydroxylysine (in yellow) at synthesis, approximately equal amounts of pyrroles and pyridinolines would result.^(^ ^22^ ^)^ Red dashed lines summarize the effect of *PLOD2*/LH2 deficiency, with fewer or no trivalent and stable divalent cross‐links forming and allysine aldol dimers the main products. The latter on tissue borohydride reduction produce the complex structures HHMD (histidinohydroxymerodesmosine) and HMD (histidinomerodesmosine; Fig. 6), and on acid hydrolysis, the artifact HHL (histidinohydroxylysinonorleucine; see Eyre and colleagues^(^ ^29^ ^)^ for details). We speculate that the material strength and toughness of the collagen as a mineralized bone composite are impaired by the labile nature of the dermis‐like cross‐linking.

In summary, the results show that LH2 deficiency almost completely prevents hydroxylation of lysine residues at telopeptide but not triple‐helical cross‐linking sites of bone type I collagen. This results in a shift in the patient's bone from the divalent keto‐imine and trivalent pyrrole and pyridinoline cross‐links of normal bone to the divalent allysine aldol cross‐links normally seen in skin not bone (Fig. 9).

## Discussion

### Clinical and genetic discussion

BS is a recessively inherited and clinically heterogeneous condition that results from biallelic pathogenic variants in either *PLOD2* (BS2) or *FKBP10* (BS1). Homozygous or compound heterozygous pathogenic variants in *PLOD2* have been shown to result in a range of clinical severities from lethal to moderate, with and without congenital contractures, even between siblings with compound heterozygosity for the identical *PLOD2* mutations.^(^ ^37^ ^)^ BS1 caused by *FKBP10* mutations also shows a broad diversity in clinical severity and phenotypic features, with and without joint contractures, even between siblings sharing identical mutations.^(^ ^20^, ^37^ ^)^

The present findings establish that bone from a patient with BS2, caused by compound heterozygous *PLOD2* mutations, has telopeptide lysine underhydroxylation and a cross‐linking abnormality that phenocopies that of BS1 bone caused by functionally null *FKBP10* mutations.^(^ ^20^ ^)^ This supports the evidence that lysyl hydroxylase‐2b encoded by *PLOD2* forms a complex with FKBP65^(^ ^16^, ^18^ ^)^ encoded by *FKBP10*, which is required for normal collagen cross‐linking in bone and certain other skeletal tissues.

The compound heterozygous mutations identified in the patients studied here have different effects on gene expression. One creates a premature termination codon that is predicted to result in nonsense‐mediated decay of *PLOD2* transcripts and no protein. The other converts a highly conserved proline in the active site region of the enzyme that could potentially ablate function. All reported BS2 causing mutations in *PLOD2* are either biallelic missense or compound heterozygous mutations.^(^ ^14^, ^37^, ^38^, ^39^, ^40^, ^41^, ^42^ ^)^ At least one of the two alleles in all BS2 cases has a missense mutation in the C‐terminal region that contains the enzymatic site for hydroxylation of telopeptide lysyl residues.^(^ ^38^ ^)^

It is reported that *Plod2*‐null mice do not survive beyond embryonic day 12,^(^ ^12^ ^)^ and suspected human cases with homozygous nonsense or frame‐shift mutations that were lethal in utero are known.^(^ ^38^ ^)^ Therefore, it seems likely that completely null alleles are lethal in mammals. In *Plod2*‐null zebrafish the phenotype presents features consistent with the bone and muscle/tendon pathology of BS but also with evidence of notochord and cartilage developmental abnormalities, suggesting a type II collagen defect.^(^ ^24^ ^)^ Currently, we do not know which lysyl hydroxylase variant is responsible for collagen type II telopeptide lysine hydroxylation; we suspect it must be a product from *PLOD2*, presumably LH2a or LH2b.

### Pyr cross‐links

Collagen in normal human bone has a distinctive cross‐linking chemistry that includes a Pyr content in the range of 0.25 to 0.35 mole/mole of collagen with a ratio of HPyr:LPyr in the range of 2:1 to 4:1.^(^ ^43^ ^)^ Pyrrole cross‐links have a similar or somewhat higher content in mature human bone as Pyrs,^(^ ^22^ ^)^ and the divalent keto‐imine and aldimine cross‐link precursors of Pyrs and pyrroles have higher contents than the trivalent cross‐links in young adult bone.^(^ ^43^ ^)^ Collagen in the *PLOD2*‐mutant bone was striking in its lack of Pyr cross‐links (1/40 the content of normal bone). The reversed ratio of HPyr:LPyr ratio (0.3:1 vs. 3:1) in the small amount of Pyr present may reflect a preferred reaction chemistry in which the few hydroxylysine aldehydes add preferentially to a neighboring lysine in the polymer rather than hydroxylysine or glycosylated hydroxylysine—resulting in more LPyr than HPyr. It is notable that *FKBP10*‐mutant BS1 bone had a similar reversed HPyr:LPyr ratio (0.27:1), and a low total Pyr content (~10% of normal).^(^ ^20^ ^)^

A preferred reaction chemistry for sparse hydroxylysine aldehydes in the growing fibril to form LPyr seems more likely than underhydroxylation of helical site lysines during synthesis in the ER. From direct mass‐spectral analyses of peptides prepared from the helical‐domain cross‐linking sites (Fig. 8), we saw no evidence of a significant effect on their hydroxylation (see below).

### Lack of telopeptide lysine hydroxylation

Direct analysis of α1(I) telopeptide Hyl:Lys ratios (Table 2) showed an essentially complete absence of Hyl, consistent with the 2% of normal Pyr content of the *PLOD2*‐mutant bone. The failure to detect any linear α2(I) N‐telopeptide fragment can be explained by 100% oxidation of the telopeptide lysine by lysyl oxidase and ensuing intramolecular α1(I)‐α2(I) aldol dimerization, consistent with previous results from *FKBP10*‐mutant human OI bone.^(^ ^20^ ^)^ The SDS‐PAGE chain profile and increased acid extractability of the bone collagen support this conclusion.

### Helical‐domain cross‐linking lysines

In normal bone about a third of both K87 and K930/933 cross‐linking sites form stable links to hydroxylysine aldehydes in fibrils.^(^ ^22^ ^)^ This means that analysis of protease‐digested peptides that had avoided cross‐linking could under‐ or over‐estimate the hydroxylation levels at synthesis, if cross‐link formation favored lysine over hydroxylysine for example. This could be significant when comparing mutant with normal bone since labile bond‐forming lysine aldehydes predominate in *PLOD2*‐mutant bone. In other words, for *PLOD2*‐mutant bone the measured Hyl:Lys ratios (Table 2) should more accurately reflect ratios on collagen synthesis, whereas for normal bone they may be skewed.

There is a precedent for interplay between the various posttranslational enzyme‐modifier complexes that work on collagen chains in the ER. For instance, lysyl hydroxylase and prolyl 3‐hydroxylase complexes exhibit a codependency in their substrate activities. Mice null for *P3h3* or *Sc65* (which encodes the essential cosubunit of *P3h3*) have underhydroxylated helical‐domain cross‐linking lysines in their skin and other tissue collagens.^(^ ^33^, ^44^ ^)^ Mice null for *P3h1* or *Crtap* (which encodes the essential cosubunit of *P3h1*) have overhydroxylated telopeptide lysines.^(^ ^1^, ^33^ ^)^ The mechanisms are unclear but are likely to involve interactions between the various modifying complexes that bind to growing procollagen chains in the ER before final triple‐helix folding and transport to the Golgi.

### Role of FKBP65 protein in regulating LH2 activity and lysine hydroxylation

The clinical similarity between patients with biallelic null *FKBP10* mutations and biallelic *PLOD2* mutations, as originally suggested, indicates that FKBP65 facilitates LH2b hydroxylase action on collagen type I telopeptides. We now know that FKBP65 binds to LH2 isoforms^(^ ^18^ ^)^; together they are part of a chaperone complex with HSP47 and BiP in the ER.^(^ ^16^ ^)^ There is recent evidence also that FKBP65 is necessary for LH2b dimerization to form the active hydroxylase.^(^ ^17^, ^18^ ^)^ The same study^(^ ^17^, ^18^ ^)^ also showed that LH2a has no telopeptide hydroxylase activity, and that FKBP65 does not promote LH1 or LH3 dimerization and activation. Therefore, based on these and other findings, LH2b appears to be the sole source of hydroxylating activity for type I collagen telopeptide lysines. We rule out LH3 because mutations in the LH3 gene cause a connective tissue disorder with an effect on helical‐domain hydroxylysine glycoside but not Pyr (telopeptide hydroxylysine) formation^(^ ^44^, ^45^ ^)^ and that cartilage type II collagen telopeptide lysines from Plod2 null zebrafish^(^ ^24^ ^)^ were not hydroxylated (unpublished findings, Charlotte Gistelinck, MaryAnn Weis, and David R Eyre).

How then can we explain why cartilage type II collagen has normal telopeptide lysine hydroxylation and Pyr formation in *PLOD2*‐ and *FKBP10‐*mutant forms of BS if LH2b is the only candidate enzyme? Perhaps, the best explanation is that chondrocytes (and ligament cells based on Bank's and colleagues’^(^ ^19^ ^)^ original tissue Pyr data from a BS1 case) express a different chaperone than FKBP65 to fold, dimerize, and activate LH2b. We can then speculate that in normal osteoblasts the active form of LH2b is a heterodimer, for example, of LH2b‐LH1 (which has been shown to form in vitro^(^ ^18^ ^)^), that requires FKBP65 to fold into an active dimer, whereas in normal and BS2 chondrocytes and ligament cells, the active form of LH2b is a homodimer that requires a different FKBP chaperone for folding and activity. The closest homolog to FKBP65 is FKBP60/*FKBP9*), which notably is expressed in the ER and in cartilage (http://www.informatics.jax.org/marker/MGI:1350921). This can explain the findings if all BS2‐causing allelic mutations in *PLOD2* are missenses that do not prevent LH2b nascent protein from being expressed in the ER but that in osteoblasts, FKBP65 is unable to fold the chains into an active dimer. In contrast, in chondrocytes and ligament cells this explanation predicts that an alternative FKBP chaperone can fold active LH2b homodimers from such missense allelic variants, so causing BS2. It is also notable that in the *PLOD2*‐mutant bone collagen, α1K930 is heavily underhydroxylated as it is normally in human bone collagen (Fig. 8). This can be explained if the missense mutant allelic product can still form a heterodimer with LH1 in the rough ER, and so effectively knock down LH1 activity but not have any LH2b activity.

There is support for such a mechanism based on the effects on bone collagen cross‐linking of biallelic null mutations in *PLOD1* that cause Ehlers‐Danlos syndrome (EDS) type VI. In EDS VI, helix domain lysines are highly underhydroxylated, yet levels of Pyr and divalent cross‐links that form from telopeptide hydroxylysine aldehydes are higher than normal in bone^27^ (unpublished findings, David R Eyre). If LH2b forms homodimers in the absence of LH1, this could explain the higher telopeptide lysine hydroxylation by osteoblasts.

In summary, the findings establish a common mechanism by which certain mutations in *PLOD2* or null mutations in *FKBP10* result in phenocopied abnormal cross‐linking of bone collagen and BS. This knowledge and that of other gene defects, which cause OI and affect bone collagen cross‐linking,^(1)^ will help in defining how normal osteoblasts regulate the unique cross‐linking chemistry of bone collagen, which we believe has evolved to optimize the packing relationship between collagen molecules and mineral crystallites in fibrils for resisting fracture.

## Disclosures

All authors state that they have no conflict of interest.

## Author Contributions

**Charlotte Gistelinck:** Conceptualization; data curation; formal analysis; methodology; validation; visualization; writing‐original draft; writing‐review and editing. **MaryAnn Weis:** Conceptualization; data curation; formal analysis; investigation; methodology; project administration; supervision; validation; visualization; writing‐original draft; writing‐review and editing. **Jyoti Rai:** Data curation; formal analysis; investigation; methodology; validation; writing‐original draft; writing‐review and editing. **Ulrike Schwarze:** Conceptualization; formal analysis; methodology; validation; writing‐original draft; writing‐review and editing. **Dmitriy Niyazov:** Conceptualization; data curation; investigation; validation; writing‐review and editing. **Kit Song:** Conceptualization; data curation; writing‐review and editing. **Peter Byers:** Conceptualization; data curation; formal analysis; investigation; validation; visualization; writing‐review and editing. **David Eyre:** Conceptualization; data curation; formal analysis; funding acquisition; investigation; methodology; project administration; resources; supervision; validation; visualization; writing‐original draft; writing‐review and editing.
